# Material damage to multielectrode arrays after electrolytic lesioning is insignificant

**DOI:** 10.7554/eLife.106452

**Published:** 2026-06-19

**Authors:** Alice Tor, Stephen E Clarke, Iliana E Bray, Paul Nuyujukian

**Affiliations:** 1 Electrical Engineering Department, Stanford University Stanford United States; 2 Bioengineering Department, Stanford University Stanford United States; 3 Neurosurgery Department, Stanford University Stanford United States; 4 Wu Tsai Neurosciences Institute, Stanford University Stanford United States; University of California, Davis United States; University of Oxford United Kingdom

**Keywords:** electron microscopy, multielectrode array, electrolytic lesion, Rhesus macaque

## Abstract

The quality of stable long-term recordings from chronically implanted electrode arrays is essential for experimental neuroscience and brain-computer interfaces. This work uses scanning electron microscopy (SEM) to image and analyze eight 96-channel Utah arrays previously implanted in motor cortical regions of four subjects (subject H = 2242 days implanted, F = 1875, U = 2680, C = 594), providing important contributions to a growing body of long-term implant research leveraging this imaging technology. Four of these arrays have been used in electrolytic lesioning experiments (H = 10 lesions, F = 1, U = 4, C = 1), a recently developed electrolytic perturbation technique demonstrated compatible with continued neuroelectrophysiology using small direct currents. Previously, our group showed that electrolytic lesioning can be used as a technique to create regions of controlled neuron loss without significantly changing recording quality (Bray, Clarke et al., 2024). Here, by surveying physical damage such as biological debris and material deterioration, we show that electrolytic lesioning causes no statistically significant material damage to the implanted electrode arrays. In addition to surveying physical damage, such as biological debris and material deterioration, this work also analyzes whether electrolytic lesioning created damage beyond what is typical for these arrays. These findings also indicate that there are no statistically significant differences between the damage observed on normal electrodes versus those used for electrolytic lesioning, yielding no evidence that electrolytic lesioning significantly affects the material quality of chronically implanted electrode arrays. Finally, this work also includes the largest collection of single-electrode SEM images for previously implanted multielectrode Utah arrays, spanning 11 different intact arrays and one broken array. As the clinical relevance of chronically implanted electrodes with single-neuron resolution continues to grow, these images may be used to provide the foundation for a larger public database and inform further electrode design and analyses.

## Introduction

In the past two decades, the advancement of implanted electrode arrays has drastically shifted the field of neuroscience from theories based on individually recorded neurons to those based on the systems-level activity of neuron populations (Cunningham and Yu, 2014; Vyas et al., 2020; Ebitz and Hayden, 2021). Specifically, the 96-channel Utah array (Blackrock Neurotech, Salt Lake City, UT) has been used to make pivotal discoveries in motor systems neuroscience and develop clinically impactful brain-computer interfaces (Maynard et al., 1997; Serruya et al., 2002; Gilja et al., 2012; Churchland et al., 2012; Gallego et al., 2020; Vyas et al., 2020). These electrode arrays are widely used in neuroprosthetics research for patients with motor disorders and have been used for a variety of purposes, such as movement and speech decoding (Gilja et al., 2015; Willett et al., 2023). Much of this work in humans relies on the translation of results discovered in non-human primates (NHPs), a crucial animal model in neuroscience research (Roelfsema and Treue, 2014; Mitchell et al., 2018). One useful application of these arrays in animal studies is microstimulation and electrolytic lesion experiments (Tehovnik et al., 2006). Electrolytic lesioning results in small, controllable, and precise amounts of neuron loss. This makes it a helpful technique to study damage to targeted areas of the brain, serving as a causal tool for basic scientific inquiry and as a model for clinical injury-based neuron loss events at a small scale.

Electrolytic lesioning has been experimented with since the 19th century (Kline, 2007), and methods have been researched in many species, including rodents, cats, dogs, monkeys, and humans (Horsley and Clarke, 1908; Sweet, 1953; Chen et al., 2009). In electrolytic lesion experiments, researchers pass current through implanted electrodes in order to mark the position of electrodes or create lesions in brain tissue (Chen et al., 2009; Townsend et al., 2002). These electrolytic lesions can be created using one electrode (unipolar) or by using two electrodes (bipolar) (Sweet, 1953; Chehrazi and Collins, 1981). In particular, recent work has established a novel lesioning technique to create consistent, controllable electrolytic lesions in NHPs using already-implanted Utah arrays, without damaging the ability to record from the array (Bray et al., 2024). However, while recording capabilities seem to remain stable, the physical effect of electrolytic lesioning on the recording array needs further characterization.

Previous studies have shown temporally decreasing performance of collected signals from long-term implanted arrays, thought to be a consequence of the immune response, mechanical movement of the array relative to surrounding tissue, and physical degradation of the array (Suner et al., 2005; Simeral et al., 2011; Downey et al., 2018). This is also reflected in studies that specifically examine the damages that occur to electrode arrays while they are implanted in NHPs (Chestek et al., 2011; Chen et al., 2023). In particular, the use of scanning electrode microscopy (SEM) has been used to visually examine and analyze the effect of neural implantation on arrays with extremely high definition (Bjånes et al., 2024; Chen et al., 2023; Patel et al., 2023; Barrese et al., 2016; Woeppel et al., 2021). These studies, which span both NHPs and humans, provide direct evidence that the observable physical damage incurred by implanted arrays has direct impact on the array’s performance.

The Utah array is a silicon-based array manufactured with several layers of different materials (Yi et al., 2022). Silicon electrodes are first etched out of a glass and silicon base. Each individual electrode is then metallized at the tip. Finally, a layer of parylene C coats all but these metallized tips to help isolate and insulate each electrode (Campbell et al., 1991; Bhandari et al., 2010). Both in vitro and in vivo testing of electrode arrays revealed environmental damage to these materials, such as cracking, textural defects, and degradation in response to the brain’s temperature and salinity (Caldwell et al., 2020; Barrese et al., 2013). The immune response of the brain also damages the electrodes due to effects like glial scarring (gliosis) and inflammation (Polikov et al., 2005; Barrese et al., 2013; Salatino et al., 2017). This damage may be exacerbated by the surgical techniques used during implantation, which include pushing the electrode array into cortex and tethering the implant to the skull (Polikov et al., 2005; Biran et al., 2007; Potter et al., 2012). These results are confirmed in the previously mentioned SEM analyses (Barrese et al., 2016; Woeppel et al., 2021; Chen et al., 2023; Patel et al., 2023; Bjånes et al., 2024).

Additionally, the Utah array is available with both platinum and iridium oxide-based coatings. Aggressive electrical stimulation is known to dissolve platinum-based electrodes (Shepherd et al., 2021; Shah et al., 2024). Other studies have shown iridium oxide to be more resistant to stimulation-related damage, but not completely insusceptible (Cogan et al., 2004; Negi et al., 2010; Chen et al., 2014; Bjånes et al., 2024). However, the physical electrode damage associated with electrolytic lesioning remains to be studied.

This work comprises images of 11 different multielectrode Utah arrays (ten 96-channel, one 64-channel). Of these imaged arrays, eight arrays chronically implanted in regions of the motor cortex for varying amounts of time were analyzed further (Monkey H = 2242 days, F = 1875, U = 2680, C = 594). Four out of eight of these arrays were used to perform electrolytic lesioning experiments (*n* = 4 monkeys; H = 10 lesions, F = 1, U = 4, C = 1). Additionally, the image set includes images of a broken 96-channel Utah array, which was shattered during explant but remained intact while implanted. This set of images represents the largest publicly available collection of high-quality, single-electrode SEM images of explanted multielectrode Utah arrays. A total of 938 individual electrodes are available to view, along with 11 additional images from the broken array. Specific details for each imaged array are available in Supplementary file 1.

## Results

All collected images, along with their associated scores, are available in interactive Video 1. Each of the 12 arrays and individual electrodes may be selected and viewed. Representative electrodes for each category and damage score, as well as electrodes used for lesioning, are also viewable for each array in an additional drop-down menu when available. Rough anatomical layouts of the array’s implant location are also included. Electrode numbering maps are available in Video 1—source data 2.

**Video 1.** Interactive display: Video demonstrates how to use the interactive display of all captured scanning electron microscopy (SEM) array images, available through the provided link. Once an array has been selected, a diagram of each array’s anatomical position (derived from surgical drawings and notes) appears, along with an SEM image of the array. All array images are displayed with the wire bundle to the right side and with electrode tips facing the viewer. Specific electrodes may be selected from the array image; SEM images of each electrode and their scores in each of the five damage categories will appear along with any additional notes. Furthermore, examples of specific scores for each damage type are selectable through an additional drop-down menu for each array. Similarly, electrodes used for electrolytic lesioning are also selectable through an additional drop-down menu for each array. Video 1—source data 1.Tutorial video for interactive display. Video 1—source data 2.Numbering layout of the electrodes as imaged (pins facing outwards, towards the reader).Wire bundle is arranged to the right. Each radial section of the array is also color-coded and labeled. R1 refers to the innermost core electrodes of the array, R2 refers to the next outer ring of the array, and so on.

**Figure 1.**
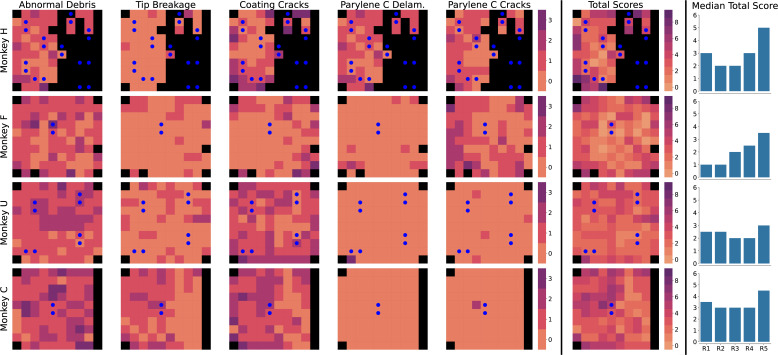
Heatmaps of damage scores (0–3) across the five identified types of damage and across the four imaged lesioning arrays. Electrodes are displayed using the orientation in Video 1 (electrode tips facing viewer, wire bundle on the right). Second-to-rightmost column displays summed damage scores for each array across the five types of damage. Electrodes used for electrolytic lesioning are denoted with blue dots. Median summed scores for each radial section of the array are plotted in the bar charts to the right of the heatmaps. Ring layout and numbering information are available in Video 1—source data 2. Unwired electrodes (electrodes not wire bonded at time of manufacture) and electrodes with shank fractures are ignored and displayed in black, as they are not scored.

The first set of analyses specifically considers the four arrays used in electrolytic lesioning experiments. Scores for each damage category, along with total scores, are visualized as heatmaps in Figure 1. Median total scores for each radial section of the array show that the outermost electrodes tend to have the greatest total observed damage across all four arrays. Generally, electrode damage decreases when moving to the center of the array. Shank fractures tend to be spatially close on each array, suggesting that shared mechanical trauma caused these sections of fracture. As mentioned in the Methods section, these fractures may have reasonably occurred during explantation and handling, as no diminished recording performance of these fractured electrodes was observed while the array was actively implanted. Additional heatmaps are available for the four additional arrays implanted in NHP but not used for lesioning in Figure 1—figure supplement 1.

**Figure 1—figure supplement 1.**
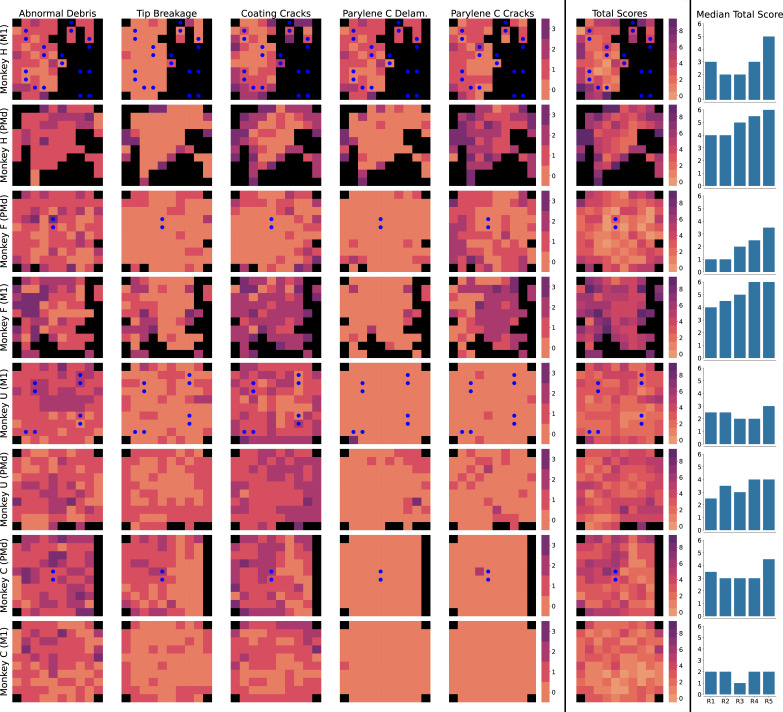
Heatmaps of damage scores (0–3) across the five identified types of damage and across all eight intact, imaged non-human primate (NHP) arrays. Electrodes are displayed using the orientation in Video 1 (electrode tips facing viewer, wire bundle on the right). Second-to-rightmost column displays summed damage scores for each array across the five types of damage. Electrodes used for electrolytic lesioning are denoted with blue dots. Median summed scores for each radial section of the array are plotted in the bar charts to the right of the heatmaps. Ring layout and numbering information is available in Video 1—source data 2. Unwired electrodes (electrodes not wire bonded at time of manufacture) and electrodes with shank fractures are ignored and displayed in black, as they are not scored.

**Figure 2.**
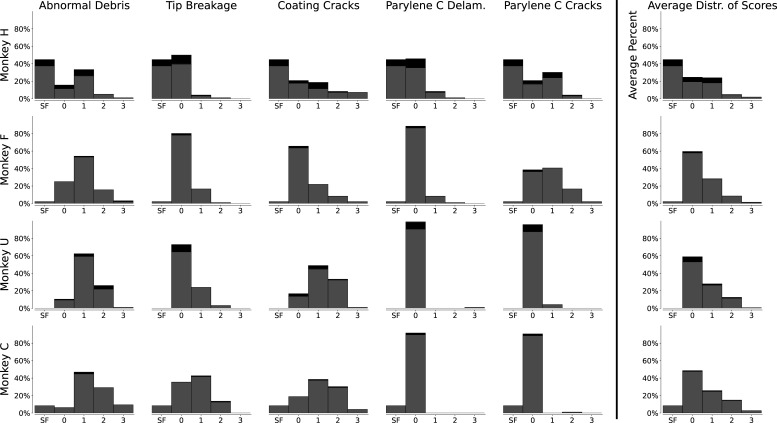
Stacked histograms of damage scores (0–3) across the five identified types of damage and across the four imaged lesioning arrays. Gray indicates normal electrodes, and black indicates lesioning electrodes. The rightmost column displays the average distribution of damage scores across the five types of damage. Electrodes with shank fractures (SF) are ignored, as they are not scored.

Histograms of damage scores in each category, along with average score distributions per array, are shown in Figure 2. For each category, histograms of electrodes used for lesioning (black) are stacked on top of histograms of normal electrodes (gray). Additionally, the average distribution of scores is shown for each array. These plots indicate that there is no observable difference in the distribution of scores given to electrodes used for lesioning versus those not. Additional histograms are available for the four additional arrays implanted in NHP but not used for lesioning in Figure 2—figure supplement 1.

**Figure 2—figure supplement 1.**
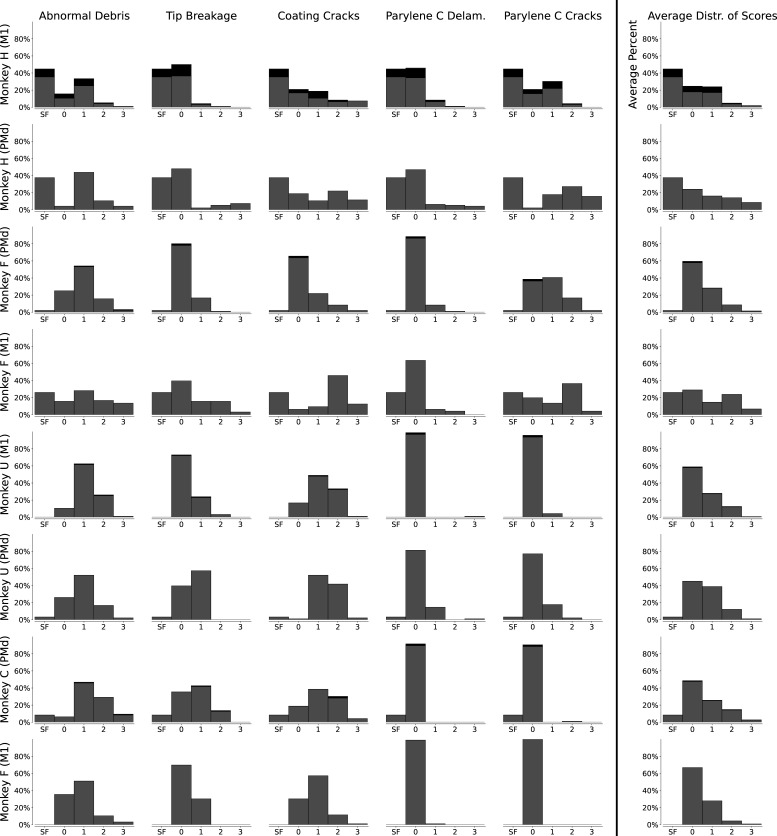
Stacked histograms of damage scores (0–3) across the five identified types of damage and across all eight intact, non-human primate (NHP) imaged arrays. Gray indicates normal electrodes, and black indicates lesioning electrodes. Rightmost column displays the average distribution of damage scores across the five types of damage. Electrodes with shank fractures (SF) are ignored, as they are not scored.

The Shapiro–Wilk test for normality returned a significant p-value for almost all sets of scores for each array and type of damage. Similarly, the Shapiro–Wilk test for normality returned a significant p-value for almost all sets of scores for each array and type of damage when split into populations used and not used for lesioning, but was not performed where electrode count was too small to carry out the test (C and F; lesioning electrodes = 2). In the majority of cases that returned an insignificant p-value, certain types of damage were not present on the array and thus scores were uniformly zero (Monkey U’s M1 tip breakage, parylene C delamination, and parylene C cracking scores; Monkey C’s PMd parylene C delamination scores and M1 parylene C cracking scores). In all other cases where the Shapiro–Wilk test returned an insignificant p-value (Monkey H’s total damage scores on lesioning electrodes, Monkey U’s coating cracks scores), low electrode counts may reduce the statistical power of the test (H = 18 lesioning electrodes, U = 8). This indicates that scores are largely not normally distributed and that nonparametric statistical tests should be used to evaluate differences between populations. All Shapiro–Wilk test p-values are available in Supplementary file 2.

For each category of damage, as well as the total damage score, no statistically significant difference was identified between lesioning and non-lesioning electrodes on the same array. Similarly, for each category of damage, as well as the total damage score, no statistically significant difference was identified between lesioning and non-lesioning electrodes pooled across all four arrays. This indicates that the electrode populations used for lesioning and those not used for recording only do not have statistically different sample means or variances, suggesting they are samples of the same underlying population. These p-values are included in Supplementary file 3. However, again, it is important to note that these tests done on individual arrays have limited power due to low electrode counts (H = 18 lesioning electrodes, F = 2, U = 8, C = 2).

This next set of analyses compares entire population statistics of all eight NHP arrays. Both the Mann–Whitney *U* and Levene tests resulted in inconsistently significant/non-significant p-values when comparing lesioning arrays against non-lesioning arrays within the same implanted animal. This indicates inconsistency in overall distribution and variance of data. This suggests that there are likely large differences between the amount and intensity of material damage dealt to implanted arrays even when implanted in the same subject. These results do not determine whether or not electrolytic lesioning experiments contribute to the magnitude of these differences, but the inconsistency in the significance of the above results may indicate that other factors, such as the brain’s varied immune response, differences in mechanical forces, and disparities in external handling, may play a large role in damage to these arrays. These p-values are included in Supplementary file 4.

**Figure 3.**
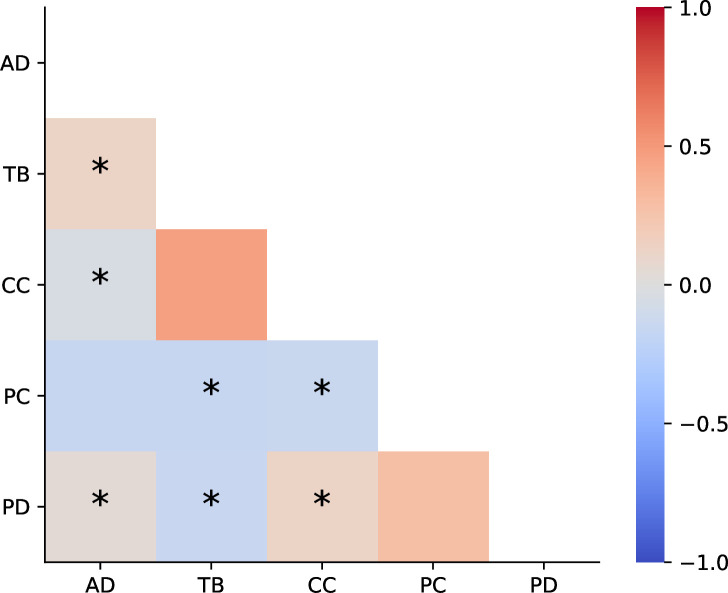
Correlation plot (Pearson’s R) across the five different damage types. AD = abnormal debris, TB = tip breakages, CC = metal coating cracks, PC = parylene C cracks, PD = parylene C delamination. Results demonstrate overall low correlation (magnitude < 0.25) across different damage types, with the exception of coating cracks and tip breakage, with a correlation coefficient of 0.47. Test values with Bonferroni-corrected p < 0.05 are displayed with asterisks. Raw p-values are separately available in Supplementary file 2. Electrodes with shank fractures are ignored, as they are not scored.

The Pearson correlation matrix across all four arrays for the five different damage types is shown in Figure 3. This plot demonstrates that there is overall low pairwise correlation between each type of damage, with the exception of coating cracks and tip breakage, which display moderate positive correlation ($r=0.47$, Bonferroni-corrected $\rm p < 0.05$). These results align with previous comparisons on the distribution of damage scores, which showed that damage scores tended to vary across the five different types. Additional correlation plots for each of the five array layout rings are available in Supplemental Materials. Raw $r$ and p-values for each test are also available in Supplementary file 5.

**Figure 3—figure supplement 1.**
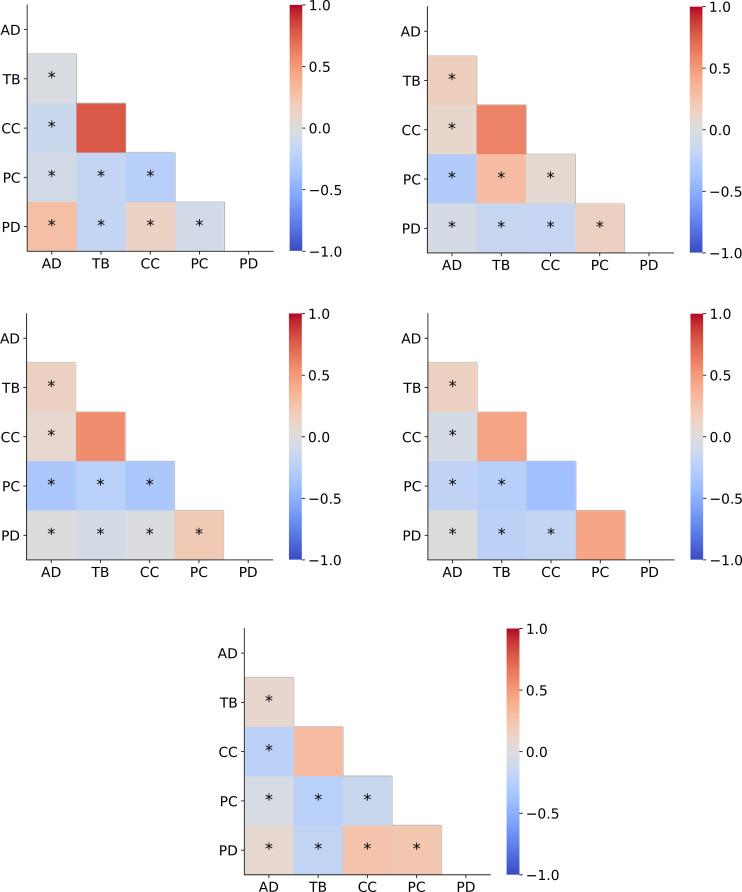
Correlation plots (Pearson’s *R*) for each of the five rings across all four imaged lesioning arrays. (Row 1, right to left: R1 and R2; row 2: R3 and R4; bottom row: R5). Test values with Bonferroni-corrected p < 0.05 are displayed with asterisks. Raw $r$ and p-values are separately available in Supplementary file 5.

**Figure 3—figure supplement 2.**
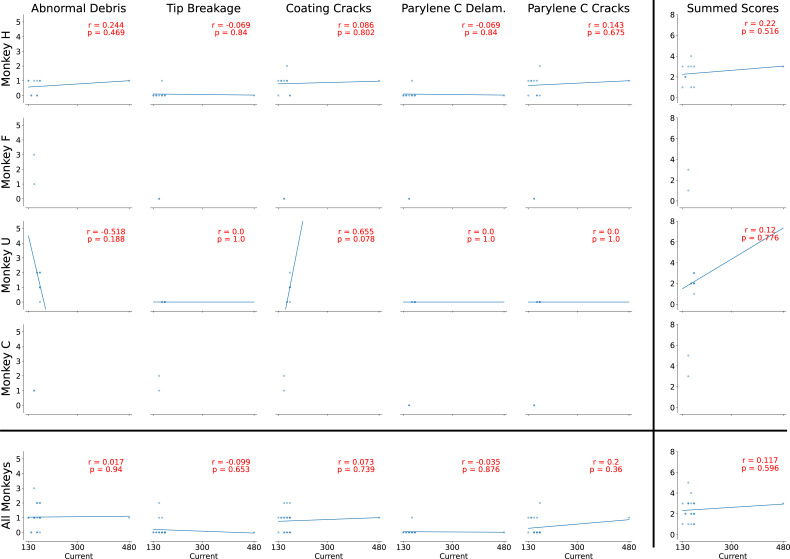
Scatterplots of the lesion electrodes’ damage scores over the target current for each lesion. A best-fit line is also plotted along with regression results. In cases where only one target current was used (ie. subjects with only one lesion), linear regression was not performed. No best-fit lines were statistically significant at p = 0.05.

**Figure 3—figure supplement 3.**
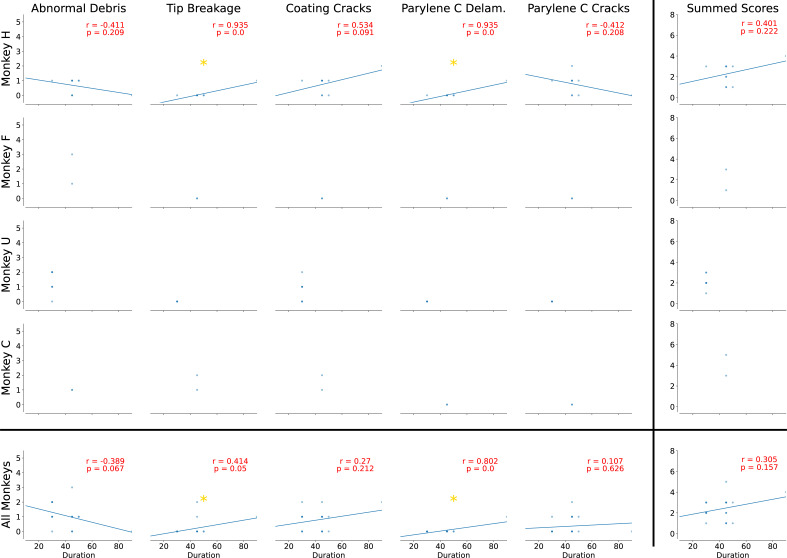
Scatterplots of the lesion electrodes’ damage scores over the target lesioning procedure duration for each lesion. A best-fit line is also plotted along with regression results. In cases where only one duration was used (ie. subjects with only one lesion, subjects with the same duration across all lesions), linear regression was not performed. Scatterplots with statistically significant lines of best fit are starred in yellow.

**Figure 3—figure supplement 4.**
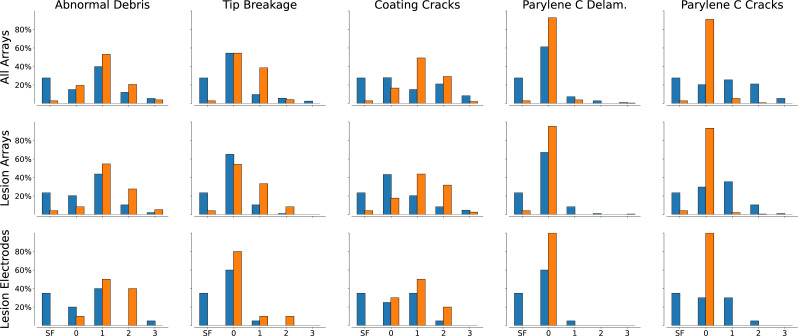
Histograms of the lesion electrodes’ damage scores, separated by array material. Blue bars are IrOx arrays and orange bars are Pt arrays. The first row shows scores across all eight scored non-human primate (NHP) arrays (*n* = 384 IrOx electrodes, *n* = 384 Pt electrodes). The second row shows scores for only the four NHP arrays used for lesioning (*n* = 192 IrOx electrodes, *n* = 192 Pt electrodes). The third row shows scores for only the subset of electrodes used for NHP lesioning (*n* = 10 IrOx electrodes, *n* = 20 Pt electrodes). Notably, it appears that platinum arrays imaged in this work generally appear to report less damage than iridium oxide arrays when considering all arrays. Additionally, the imaged platinum arrays appear to always report less parylene C damage. However, platinum arrays generally appear to report more other types of damage (abnormal debris, tip breakage, coating cracks) when used for lesioning. This supports previous literature that indicates IrOx may be more resistant to stimulation-related damage than Pt (Cogan et al., 2004; Negi et al., 2010; Chen et al., 2023; Bjånes et al., 2024). It is important to note that many other factors, such as time in tissue, explant surgery, and immune response of the specific implant subject, all impact the conclusions of this analysis.

## Discussion

Overall, these results collectively demonstrate that there is no obvious, statistically significant difference between observed damage to electrodes used versus not used for electrolytic lesioning. This indicates that any material effects potentially caused by electrolytic lesioning are indistinguishable from the typical damage seen in long-term implanted arrays. Because this method of lesioning involves passing weak direct current into the brain over relatively short time periods, its physical effects on the array electrodes are similar to alternating current stimulation. A recent study found that SEM-visible damage caused by stimulation on iridium oxide electrodes is highly variable between arrays, where one imaged array demonstrated noticeable stimulation-related damage and the other array had no such damage (Woeppel et al., 2021). Furthermore, the study found that array recording quality was not compromised by stimulation. Similarly, other work did not show significant differences in SEM-visible degradation between platinum electrodes used for stimulation and iridium oxide electrodes used for stimulation (Bjånes et al., 2024; Chen et al., 2023). All of these previous studies used different stimulation protocols: a pulse train delivered at 20–300 Hz at amplitudes 1–100 μA (Woeppel et al., 2021), a pulse train delivered at 300 Hz at amplitudes 1–210 μA (Chen et al., 2023), and an undisclosed low-current stimulation (Bjånes et al., 2024). Additionally, dissolution of electrodes due to current is most salient at aggressive levels of stimulation; one study reviewing electrode dissolution delivered variable amplitudes of current at 50 Hz for 7 hr Negi et al., 2010; another study utilized currents at least one order of magnitude greater than the above protocols (Shepherd et al., 2021). The electrolytic lesioning in this work primarily used direct currents delivered at around 150 μA for 45 s, though parameters ranged between 50–450 μA and 12–600 s. Thus, despite the differences between specific lesioning and stimulation protocols, the findings presented here are consistent with previous studies that found no unusual damage to the electrodes used for stimulation. This aligns with previous findings that electrolytic lesioning does not significantly impact the recording ability of electrodes (Bray et al., 2024).

The patterns of damage found in this work also largely reflect previous SEM analyses of explanted arrays. A prior study also found that the electrodes closest to the perimeter of the array suffered the most damage (Bjånes et al., 2024). This pattern is also visually observable in other published results (Patel et al., 2023; Woeppel et al., 2021). This is likely because the edges of the arrays serve as a physical shield for the innermost electrodes. Edge electrodes are subject to greater access to brain tissue, as well as to mechanical damage incurred during surgery and handling.

The frequency of damage types identified in this work differs from previous studies, but is not unusual. In this work, the most common damage type within arrays used for lesioning is abnormal debris (*n* = 276, 71.9%), followed by metal coating cracks (*n* = 214, 55.7%), tip breakage (*n* = 102, 26.6%), parylene C cracks (*n* = 95, 24.7%), and parylene C delamination (*n* = 48, 12.5%). On these lesioning arrays, there were *n* = 53, or 13.8%, shank fractures. Similarly, across all eight NHP arrays imaged, the most common damage type is abnormal debris (*n* = 518, 67.4%), followed by metal coating cracks (*n* = 480, 62.5%), tip breakage (*n* = 233, 30.3%), parylene C cracks (*n* = 224, 29.2%), and parylene C delamination (*n* = 60, 7.8%). Across all eight NHP arrays, there were *n* = 117, or 15.2%, shank fractures. However, a prior study found that coating cracks were the most common type of observed damage, followed closely by parylene C cracks, then tip breakage, abnormal debris, and parylene C delamination (Patel et al., 2023). While the frequency of damage types is not exactly aligned, previous work included a cleaning step during array preparation for SEM imaging; this work does not explicitly attempt to remove excess brain tissue from imaged arrays, which likely led to the relative increase we observed in abnormal debris. Additionally, variation in individual researchers’ scoring standards may also contribute to differences. As mentioned above, the overall spatial pattern of damage between these two studies is consistent. Finally, this study also found a moderate positive correlation between coating cracks and tip breakage. This reflects the fact that damage to the silicon core of an electrode often occurs in tandem with cracking and flaking of the surrounding metal coating; the silicon cannot break without also breaking its coating. This correlation is visible in the collected images presented in Video 1.

This work analyzed high-definition SEM images of previously implanted electrode arrays used for electrolytic lesioning. Across all eight arrays previously implanted in NHPs, damage disproportionately occurred to the outer edges of arrays. Additionally, no statistically significant difference was found between the damage experienced by normal and lesioning electrodes within the same array. Furthermore, statistical testing between arrays used and not used for lesioning experiments did not indicate consistent, significant differences. Finally, this work also presents the largest publicly available set of SEM images of explanted arrays, consisting of 11 different multielectrode Utah arrays (ten 96-channel, one 64-channel) and one broken 96-channel array. These results from this dataset align with previous SEM studies of chronically implanted arrays used for both recording and stimulation, while providing further evidence for electrolytic lesioning as a safe and useful technique for experimental neuroscience.

## Methods

### Electrolytic lesioning

All lesions were performed following previously described procedures (Bray et al., 2024). To summarize, each lesion is created by passing around 150 μA of direct current for approximately 45 s between two adjacent electrodes (one anode and one cathode) on the array. Thus, each lesion is bipolar, not unipolar, and is produced without a pulse/frequency-based protocol. Exact parameters, ranging between 50–450 μA and 12–600 s for each lesion, are available in Supplementary file 6.

### Array preparation

Nine explanted NHP arrays were utilized for this study. Monkey H, F, and C were each implanted with two 96-channel Utah arrays in M1 (primary motor cortex) and PMd (dorsal premotor cortex). H’s M1 array was used for electrolytic lesioning experiments. F’s and C’s PMd arrays were used for electrolytic lesioning experiments. Monkey U was implanted with three 96-channel arrays; two in M1, and one in PMd. U’s lateral M1 array was used for electrolytic lesioning experiments. U’s medial M1 array was not used for recording, and was damaged during extraction. Monkey H’s arrays were implanted for 2242 days (electrolytic lesions on days 2088, 2129, 2136, 2164, 2172, 2180, 2187, 2192, 2221, and 2228). Monkey F’s arrays were implanted for 1875 days (electrolytic lesion on day 1875). Monkey U’s arrays were implanted for 2680 days (electrolytic lesions on days 1215, 1250, 1264, and 1286). Monkey C’s arrays were implanted for 594 days (electrolytic lesion on day 304). To produce each electrolytic lesion, current was passed between two electrodes on the array. Further details on each monkey’s arrays, as well as any additional imaged arrays, are available in Supplementary file 1. All monkeys were male rhesus macaques. Further details on animal and lesioning procedures are available in a prior report (Bray et al., 2024). All animal procedures and protocols were approved by the Stanford University Institutional Animal Care and Use Committee (#D16-00134).

All arrays used in this work were 10 × 10 multielectrode Utah arrays (Blackrock Neurotech, Salt Lake City, UT). Monkey C was implanted with an array with electrode shank lengths of 1.5 mm, while Monkeys H, F, and U were implanted with arrays with electrode shank lengths of 1 mm. Monkey U and C’s arrays were manufactured with an iridium oxide metal coating, while H and F’s arrays were manufactured with platinum coatings.

Arrays were first soaked in either a 0.9% saline or 10% formalin solution immediately following explant surgery, then stored separately in either water, formalin, ethanol, or dry in a closed container. Prior to imaging, all arrays were allowed to dry without any further cleaning or preparation.

### Scanning electron microscopy

SEM images were collected using a Hitachi TM4000+ Tabletop SEM (Hitachi Ltd, Tokyo, Japan). Arrays were mounted on PELCO SEMClip Pin Mounts (Ted Pella, Redding, CA) using conductive copper tape, carbon tape, and included clip mounts. Arrays were not sputter-coated for imaging. Due to the amount of organic material left on the arrays, images were collected in low-vacuum mode to avoid distortion from accumulated electrical charge of the non-conductive material. Full array images were collected with the arrays flat on the mount surface, while single-electrode images were collected with the arrays positioned at an approximate 45°angle. Most images were collected using a mixed backscatter/secondary electron (BSE/SE) mode with a low accelerating voltage of 5 kV, although modes and voltage values were adjusted for certain electrodes to optimize image clarity. Specific settings for each collected image are included in Video 1.

### Damage scoring

A prior study categorized damage observable with SEM into six distinct types: abnormal debris, tip breakage, coating cracks (cracking of the metal coating around the silicon core of an electrode), parylene C cracks, parylene C delamination, and shank fracture (Patel et al., 2023). Similarly, damage to the arrays in this study were scored in five categories: abnormal debris (AB), silicon tip breakage (TB), platinum coating cracks (CC), parylene C coating cracks (PC), and parylene C coating delamination (PD). Each recording electrode was scored from 0 to 3 in five different categories, as described above. Electrodes with shank fractures (SF) were not scored, as these fractures may have reasonably occurred during explantation and handling of the arrays separate from the damage incurred while in the brain. Each electrode was scored between 0–3, where 0 indicated no visible effects, 1 indicated visible effects but likely no impact on recording ability, 2 indicated possible impact on recording ability, and 3 indicated likely impact on recording ability. Scores were given without prior knowledge of which electrodes had been used for electrolytic lesioning. Although this scoring system is subjective and based on visual observation, all images used to compute these scores are publicly available in Video 1 for independent evaluation.

### Statistical tests

First, the Pearson’s correlation coefficient was calculated between each subcategory of damage to determine whether any types of damage were related. To explore potential spatial relationships, this analysis was repeated for each radial ring of electrodes, as shown in Figure 3—figure supplement 1.

Statistical significance was also assessed between electrodes used in the electrolytic lesion experiments versus those which had not. The Shapiro–Wilk test indicated that scores did not follow a normal distribution; therefore, nonparametric methods such as the Mann–Whitney *U* test and the Levene test were used to compare the underlying statistics of each array’s electrode population. The null hypothesis of the Mann–Whitney *U* test is that sample distributions of two sets of scores are the same. The null hypothesis of the Levene test is that the sample variance of two sets of scores are the same.

Similarly, statistical significance was assessed as described above between lesioning versus normal electrodes, which were pooled across all four arrays used for electrolytic lesioning. Finally, this analysis was repeated between electrode scores across pairs of lesioning and non-lesioning arrays implanted in the same subject for the same length of time.

## Data Availability

All images and data used in this analysis have been deposited at the Stanford Data Repository (https://doi.org/10.25740/dz983xf0632). All data for this manuscript are embedded into the source code of Video 1. The following dataset was generated: TorA ClarkeS BrayI NuyujukianP 2025Materials associated with Tor, et al., eLife 2025Stanford Data Repository10.25740/dz983xf0632

## Additional files

MDAR checklist

Supplementary file 1. Details and characteristics for all imaged and analyzed devices in this work.

Supplementary file 2. Shapiro-Wilk p-values.

Supplementary file 3. Mann-Whitney U and Levene p-values when comparing damage scores given to lesioning/non-lesioning electrodes.

Supplementary file 4. Mann-Whitney U and Levene p-values when comparing total damage scores given to arrays used for lesioning/non-lesioning.

Supplementary file 5. Correlation r and p-values.

Supplementary file 6. Details for electrolytic lesions referenced in this manuscript.
