# Supplementary material for: Material damage to multielectrode arrays after electrolytic lesioning is insignificant
Source: eLife. 2026 Jun 19;14:RP106452. doi: 10.7554/eLife.106452 (PMC13282118; doi:10.7554/eLife.106452)
Supplement: Video 1—source data 1. [file elife-106452.zip › SEM_selector_v4.html]

Select an Array

H PMd
H M1
F PMd
F M1
C PMd
C M1
U PMd
U M1 (medial)
U M1 (lateral)
P
Agar
Control

Scored Electrode Examples

Abnormal Debris: 1
Abnormal Debris: 2
Abnormal Debris: 3
Silicon fracture: 1
Coating cracks: 1
Coating cracks: 2
Coating cracks: 3
Parylene C delamination: 1

## C M1

Click each electrode to view a close-up:

Scored Electrode Examples

Abnormal Debris: 1
Abnormal Debris: 2
Abnormal Debris: 3
Silicon fracture: 1
Silicon fracture: 2
Coating cracks: 1
Coating cracks: 2
Coating cracks: 3
Parylene C delamination: 1
Parylene C delamination: 2
Shank fracture

Lesion Electrodes

Lesion Electrode 53
Lesion Electrode 54

## C PMd

Click each electrode to view a close-up:

Scored Electrode Examples

Abnormal Debris: 1
Abnormal Debris: 2
Abnormal Debris: 3
Silicon fracture: 1
Silicon fracture: 2
Silicon fracture: 3
Coating cracks: 1
Coating cracks: 2
Coating cracks: 3
Parylene C delamination: 1
Parylene C delamination: 2
Parylene C cracks: 1
Parylene C cracks: 2
Parylene C cracks: 3
Full shank fracture

## F M1

Click each electrode to view a close-up:

Scored Electrode Examples

Abnormal Debris: 1
Abnormal Debris: 2
Abnormal Debris: 3
Silicon fracture: 1
Silicon fracture: 2
Coating cracks: 1
Coating cracks: 2
Coating cracks: 3
Parylene C delamination: 1
Parylene C delamination: 2
Parylene C cracks: 1
Parylene C cracks: 2
Parylene C cracks: 3
Full shank fracture

Lesion Electrodes

Lesion Electrode 52
Lesion Electrode 53

## F PMd

Click each electrode to view a close-up:

Scored Electrode Examples

Tip 1
Tip 2
Tip 3
Tip 4
Tip 5
Tip 6
Tip 7
Shank 1
Shank 2
Group 1
Group 2

## U M1 (medial)

This array physically rotated while implanted, resulting in poor signal quality. It was found sideways in M1 during explantation, and was broken during removal from tissue. A sampling of the broken shafts were mounted and imaged.

Click to view certain electrode tips, shanks, and groups (all are listed in drop-down menu):

Scored Electrode Examples

Abnormal Debris: 1
Abnormal Debris: 2
Abnormal Debris: 3
Silicon fracture: 1
Coating cracks: 1
Coating cracks: 2
Coating cracks: 3
Parylene C delamination: 1
Parylene C delamination: 3
Parylene C cracks: 1
Parylene C cracks: 2
Shank fracture

## U PMd

Click each electrode to view a close-up:

Scored Electrode Examples

Abnormal Debris: 1
Abnormal Debris: 2
Abnormal Debris: 3
Silicon fracture: 1
Silicon fracture: 2
Coating cracks: 1
Coating cracks: 2
Coating cracks: 3
Parylene C delamination: 3
Parylene C cracks: 1

Lesion Electrodes

Lesion Electrode 20
Lesion Electrode 21
Lesion Electrode 25
Lesion Electrode 26
Lesion Electrode 71
Lesion Electrode 72
Lesion Electrode 77
Lesion Electrode 87

## U M1 (lateral)

Click each electrode to view a close-up:

Scored Electrode Examples

Abnormal Debris: 1
Abnormal Debris: 2
Abnormal Debris: 3
Silicon fracture: 1
Coating cracks: 1
Coating cracks: 2
Coating cracks: 3
Parylene C delamination: 1
Parylene C delamination: 2
Parylene C cracks: 1
Parylene C cracks: 2
Full shank fracture

Lesion Electrodes

Lesion Electrode 11
Lesion Electrode 12
Lesion Electrode 15
Lesion Electrode 18
Lesion Electrode 25
Lesion Electrode 28
Lesion Electrode 29
Lesion Electrode 30
Lesion Electrode 43
Lesion Electrode 44
Lesion Electrode 62
Lesion Electrode 63
Lesion Electrode 67
Lesion Electrode 77
Lesion Electrode 80
Lesion Electrode 81
Lesion Electrode 85
Lesion Electrode 86

## H M1

Click each electrode to view a close-up:

Scored Electrode Examples

Abnormal Debris: 1
Abnormal Debris: 2
Abnormal Debris: 3
Silicon fracture: 1
Silicon fracture: 2
Silicon fracture: 3
Coating cracks: 1
Coating cracks: 2
Coating cracks: 3
Parylene C delamination: 1
Parylene C delamination: 2
Parylene C delamination: 3
Parylene C cracks: 1
Parylene C cracks: 2
Parylene C cracks: 3
Full shank fracture

## H PMd

Click each electrode to view a close-up:

## A

Used in initial design, testing, and calibration of lesion circuitry. Brain tissue was mimicked using an agar-based gel containing an agar (Sigma-Aldrich) and saline solution. A wide range of currents (50-500uA) and durations (30s to 10 minutes) were applied repeatedly.

Click each electrode to view a close-up:

## Control

Images of never-implanted array. Damage and debris are due to handling. One image is available per column.

Click each column to view a close-up of a single electrode in the 5th row (from top to bottom):

## P

Used in multiple lesions associated with Bray\*, Clarke\*, et al., eLife 2024. Implanted and removed from brain tissue mutiple times over weeks/months.

Click each electrode to view a close-up:
