# Supplementary material for: Material damage to multielectrode arrays after electrolytic lesioning is insignificant
Source: eLife. 2026 Jun 19;14:RP106452. doi: 10.7554/eLife.106452 (PMC13282118; doi:10.7554/eLife.106452)
Supplement: Supplementary file 1. [file elife-106452-supp1.pdf]

Supplemental Table 1:

Details and characteristics for all imaged and analyzed devices in this work.

Subject	Region	Metal	DOB	Implant Date	Explant Date	Days	Serial Num	Size	Notes
H	PMd	Pt	Dec 14 2006	Mar 17 2014	6 May 2020	2242	1024-1177	96	Missing wire bundle – layout/numbering is arbitrary
H	M1	Pt	Dec 14 2006	Mar 17 2014	6 May 2020	2242	1024-1166	96	
F	PMd	Pt	2003	Sep 8 2014	27 Oct 2019	1875	1024-1171	96	
F	M1	Pt	2003	Sep 8 2014	27 Oct 2019	1875	1024-1175	96	Missing wire bundle; previously encapsulated in fibrin
U	PMd	IrOx	Mar 22 2010	Aug 4 2017	Dec 5 2024	2680	1024-1903	96	
U	M1 medial posterior	IrOx	Mar 22 2010	Aug 4 2017	Dec 5 2024	2680	1024-1902	96	Broken during extraction
U	M1 lateral anterior	IrOx	Mar 22 2010	Aug 4 2017	Dec 5 2024	2680	1024-1905	96	
C	PMd	IrOx	May 15 2007	Mar 25 2021	9 Nov 2022	594	6250-001604	96	
C	M1	IrOx	May 15 2007	Mar 25 2021	9 Nov 2022	594	6250-001608	96	
Agar				25 Aug 2022	25 Aug 2022		1025-149	64	Implanted briefly into agar gel and used to test initial electrolytic lesioning protocols during development.
P		Pt					1024-0577	96	Used in multiple lesions associated with Bray*, Clarke*, et al., eLife 2024. Implanted and removed from brain tissue multiple times over weeks/months.
Control								96	Never-implanted array. Damage and debris are due to handling. One image is available per column.
