# Supplementary material for: Material damage to multielectrode arrays after electrolytic lesioning is insignificant
Source: eLife. 2026 Jun 19;14:RP106452. doi: 10.7554/eLife.106452 (PMC13282118; doi:10.7554/eLife.106452)
Supplement: Supplementary file 5. [file elife-106452-supp5.pdf]

Supplemental Table 5:
Bonferroni-corrected p-value is 0.005 (0.05/n=10 comparisons).

	All		R1		R2		R3		R4		R5	
Damage Types	r	p-val	r	p-val	r	p-val	r	p-val	r	p-val	r	p-val
AD/AD	1.0000	< 0.001	1.0000	< 0.001	1.0000	< 0.001	1.0000	< 0.001	1.0000	< 0.001	1.0000	< 0.001
AD/TB	0.1226	< 0.05	-0.0292	0.9178	0.1591	0.3141	0.1387	0.2521	0.1375	0.1704	0.0792	0.4268
AD/CC	-0.0443	0.4215	-0.1274	0.6509	0.0937	0.5549	0.0778	0.5221	-0.0619	0.5383	-0.2294	0.0198
AD/PC	-0.1621	< 0.005	-0.0714	0.8003	-0.2846	0.0677	-0.3252	< 0.01	-0.1970	< 0.05	-0.0499	0.6163
AD/PD	0.0437	0.4278	0.2857	0.3019	-0.0577	0.7168	-0.0018	0.9882	-0.0064	0.9492	0.0775	0.4366
TB/TB	1.0000	< 0.001	1.0000	< 0.001	1.0000	< 0.001	1.0000	< 0.001	1.0000	< 0.001	1.0000	< 0.001
TB/CC	0.4676	< 0.001	0.7802	< 0.001	0.6069	< 0.001	0.5575	< 0.001	0.4405	< 0.001	0.3100	< 0.005
TB/PC	-0.1527	< 0.01	-0.1750	0.5328	0.2901	0.0624	-0.2391	< 0.05	-0.2535	< 0.05	-0.2411	< 0.05
TB/PD	-0.1449	< 0.01	-0.1750	0.5328	-0.1614	0.3071	-0.0674	0.5792	-0.2227	< 0.05	-0.1925	0.0514
CC/CC	1.0000	< 0.001	1.0000	< 0.001	1.0000	< 0.001	1.0000	< 0.001	1.0000	< 0.001	1.0000	< 0.001
CC/PC	-0.1366	< 0.05	-0.2548	0.3594	0.0677	0.6701	-0.3252	< 0.01	-0.3775	< 0.001	-0.1396	0.1595
CC/PD	0.1203	< 0.05	0.1274	0.6509	-0.1658	0.2940	-0.0188	0.8775	-0.1760	0.0783	0.2562	< 0.01
PC/PC	1.0000	< 0.001	1.0000	< 0.001	1.0000	< 0.001	1.0000	< 0.001	1.0000	< 0.001	1.0000	< 0.001
PC/PD	0.2889	< 0.001	-0.0714	0.8003	0.1332	0.4003	0.1979	0.1005	0.4326	< 0.001	0.2292	< 0.05
PD/PD	1.0000	< 0.001	1.0000	< 0.001	1.0000	< 0.001	1.0000	< 0.001	1.0000	< 0.001	1.0000	< 0.001
