# Supplementary material for: Material damage to multielectrode arrays after electrolytic lesioning is insignificant
Source: eLife. 2026 Jun 19;14:RP106452. doi: 10.7554/eLife.106452 (PMC13282118; doi:10.7554/eLife.106452)
Supplement: Peer review history

# Editor report: eLife Assessment

**DOI:** 10.7554/eLife.106452.3.sa0

**Authors:** Xiaomo Chen

**Affiliations:** University of California, Davis United States

**Keywords:** Solid, Useful

This **useful** manuscript addresses a stability issue for long-term chronically implanted array recordings and electrolytic lesioning, which is relevant to both basic science and translational research. The authors provide a systematic scanning electron microscopy (SEM) of explanted arrays, evaluating electrode damage and sharing extensive datasets accessible through interactive plots. The strength of the evidence is **solid**, but it can be improved by performing additional analyses on complementary neurophysiology, functional, or histological data.

# Referee report: Reviewer #1 (Public review)

**DOI:** 10.7554/eLife.106452.3.sa1

Summary:

This work presents a GUI with SEM images of 8 Utah arrays (8 of which were explanted, and 4 of which were used for creating cortical lesions).

Strengths:

Visual comparison of electrode tips with SEM images, showing that electrolytic lesioning did not appear to cause extra damage to electrodes.

Weaknesses:

Given that the analysis was conducted on explanted arrays, and no functional or behavioural in-vivo data or histological data are provided, any damage to the arrays may have occurred after explantation, making the results limited and inconclusive (firstly, that there was no significant relationship between degree of electrode damage and use of electrolytic lesioning, and secondly, that electrodes closer to the edge of the arrays showed more damge than those in the center).

Overall, these results add new data and reference images to the field, although the insights that can conclusively be drawn are limited due to the low number of electrodes used and lack of in-vivo/ histological/ impedance data.

# Author comment: Author response

**DOI:** 10.7554/eLife.106452.3.sa2

**Authors:** Alice Tor, Stephen E Clarke, Iliana E Bray, Paul Nuyujukian, Brain Interfacing Laboratory

**Affiliations:** Stanford University Stanford United States; Stanford University Stanford United States; Stanford University Stanford United States; Stanford University Stanford United States; Stanford University STANFORD United States

The following is the authors’ response to the original reviews.

**Public Reviews:**

**Reviewer #1 (Public review):**

Summary:

This work presents a GUI with SEM images of 8 Utah arrays (8 of which were explanted, and 4 of which were used for creating cortical lesions).

Strengths:

Visual comparison of electrode tips with SEM images, showing that electrolytic lesioning did not appear to cause extra damage to electrodes.

Weaknesses:

Given that the analysis was conducted on explanted arrays, and no functional or behavioural in vivo data or histological data are provided, any damage to the arrays may have occurred after explantation. This makes the results limited and inconclusive (firstly, that there was no significant relationship between degree of electrode damage and use of electrolytic lesioning, and secondly, that electrodes closer to the edge of the arrays showed more damage than those in the center).

We agree insofar as we could not fully control the circumstances of each array during explantation. However, array explantation is potentially damaging, but not universally damaging, as demonstrated by some largely intact arrays in this paper. If electrolytic lesions were damaging to the array, they would be observed. All arrays examined in this paper were carefully stored as described in the paper. All analyses of this type require an explant surgery. Our conclusions remain as strong as any of the results of these analyses.

Overall, these results do not add new insight to the field, although they do add more data and reference images.

We respectfully disagree, as there is no extant SEM analysis on electrode arrays used for lesioning.

**Reviewer #2 (Public review):**

In this study, the authors used scanning electron microscopy (SEM) to image and analyze eleven Utah multielectrode arrays (including eight chronically implanted in four macaques). Four of the eight arrays had previously been used to deliver electrolytic lesions. Each intact electrode was scored in five damage categories. They found that damage disproportionately occurred to the outer edges of arrays. Importantly, the authors conclude that their electrolytic Lesioning protocol does not significantly increase material degradation compared to normal chronic use without lesion. Additionally, the authors have released a substantial public dataset of single-electrode SEM images of explanted Utah arrays. The paper is well-written and addresses an important stability issue for long-term chronically implanted array recordings and electrolytic lesioning, which is relevant to both basic science and translational research. By comparing lesioning and non-lesioning electrodes on the same array and within the same animal, the study effectively controls for confounds related to the animal and surgical procedures. The shared dataset, accessible via interactive plots, enhances transparency and serves as a valuable reference for future investigations. Below, we outline some major and minor concerns that could help improve the work.

Major concerns:

(1) Electrode impedance is a critical measurement to evaluate the performance of recording electrodes. It would be helpful if the authors could provide pre-explant and post-explant impedance values for each electrode alongside the five SEM damage scores. This would allow the readers to assess how well the morphological scores align with functional degradation.

We agree, electrode impedance is very important in determining electrode performance. However, due to the multi-year, multi-subject nature of this work, we unfortunately do not have this data.

(2) The lesion parameters differ across experiments and electrodes. It would be helpful if the authors could evaluate whether damage scores (and/or impedance changes) correlate with total charge, current amplitude, duration, or frequency.

Thank you for this recommendation. We have included additional analyses in Supplementary Materials.

**Recommendations for the authors:**

**Reviewer #1 (Recommendations for the authors):**

(1) ‘Both in vitro and in vivo testing of electrode arrays revealed environmental damage to these materials, such as cracking, textural defects, and degradation in response to the brain’s temperature and salinity [32]. The immune response of the brain also damages the electrodes due to effects like glial scarring (gliosis) and inflammation [33, 34]. This damage may be exacerbated by the surgical techniques used during implantation, which include pushing the electrode array into cortex and tethering the implant to the skull [33, 35, 36].’

In the above text, several relevant references have been left out, e.g.:

Barrese et al., 2013

Patel et al., 2023

Woeppel et al, 2021

Chen et al., 2023

Bjanes et al., 2025

Thank you for this recommendation. This section has been updated.

(2) ‘Aggressive electrical stimulation is known to dissolve platinum-based electrodes [37, 38]. Other studies have shown iridium oxide to be more resistant to stimulation-related damage, but not completely insusceptible [39, 40].’ Reference number 25 is relevant here.

Thank you for this recommendation. This section has been updated.

(3) ‘F’s and C’s PMd arrays were used for electrolytic lesioning experiments Monkey U was implanted with three 96-channel arrays; two in M1 and one in PMd.’ There seems to be a punctuation mark missing.

Thank you for this recommendation. This section has been updated.

(4) Methods: How much charge was injected via the electrodes that were used for lesioning? What current amplitudes, voltages, durations, and number of pulses were used? If more than 1 pulse was applied, what were the frequencies? Was the pulse cathode-only/ anode/only? What were the electrode impedance values at the time of stimulation? How many electrodes were used for lesioning at any given moment? How long after lesioning did the arrays remain in the tissue?

Thank you for your questions. An additional supplemental table (Supplemental Table 6) detailing specific NHP lesions parameters has been added. A summary of the lesion procedure (DC, bipolar, two electrodes at a time) has also been included in Methods. All arrays remained in the subject until explant, which ranged between hours (same-day lesion and explant) to several years. Further details on the lesioning procedure are available in citation [?]. Explant dates are available in Supplemental Table 1. Unfortunately, we do not have the impedance values at time of lesioning as this is not a measure we record frequently after implant, though we agree the data would be useful to have.

(5) Caption for Figure 1: ‘All array images are displayed with the wire bundle to the right side.’ I recommend adding this text from Figure 2 to the caption of Figure 1: ’electrode tips facing viewer’.

Thank you for this recommendation. This section has been updated.

(6) ‘Electrodes used for electrolytic lesioning are denoted with blue dots.’ Was stimulation carried out across all these electrodes simultaneously?

No, stimulation was not carried out across all electrode simultaneously. Pairs of electrodes were stimulated at the same time to create lesions. Lesions were performed on different days. We have updated our methods section to reflect this. See the Methods section and citation [?] for more details.

(7) For the control array, in Figure 1: ‘Click each column to view a close-up of the 5th row (from top to bottom) of electrodes:’ . It would be clearer to state: ’Click each column to view a close-up of a single electrode in the 5th row (from top to bottom):’.

Thank you for this recommendation. This section has been updated.

(8) Figure 2 caption: ‘Blank electrodes and electrodes with shank fractures are ignored and displayed in black, as they are not scored.’. What is a ‘blank’ electrode?

A ‘blank’ electrode is an electrode on the array that physically exists but is not wire bonded at time of manufacture to produce recordings. The corner electrodes of the Utah array are all blank electrodes. We have updated this wording to ‘unwired’ for clarity.

(9) I recommend incorporating Supplementary Figure 1 into Figure 2, so that the reader can immediately see where the rings are, without referring to the Supplementary Materials.

Thank you for this recommendation. We have chosen to keep these figures separate for stylistic reasons.

(10) Supplementary Figures: The figures should have the word ’Supplementary’ in the title, i.e., ‘Supplementary Figure X,’ not just ‘Figure X.’

Thank you for this recommendation. These captions have been updated.

(11) Throughout the results, the text is overly focused on the type of statistical test used and the p-values, e.g.: ‘When comparing lesioning and non-lesioning electrodes within the same array, each of the two nonparametric statistical tests (Mann-Whitney U-test, Levene Test) returned insignificant p-values for each category of damage as well as for total damage scores for all four arrays used in lesioning experiments.’.

To make the findings more digestible for the reader, the text should be rephrased in terms of whether the metrics being compared were significantly different or not. E.g.: ‘For each category of damage, as well as for the total damage score, no significant difference was found between electrodes that were or were not used for lesioning (either the mean or the variance of the scores).’.

Thank you for this recommendation. We have rephrased the text to reflect this note.

(12) ‘In Monkey H, the Mann-Whitney U test resulted in an insignificant p-value for coating cracks and parylene C delamination scores, while the Levene test resulted in an insignificant p-value for abnormal debris, coating cracks, and parylene C cracking scores. In Monkey F, the Mann-Whitney U test resulted in an insignificant p-value for parylene C delamination scores, while the Levene test resulted in an insignificant p-value for coating cracks, parylene C delamination, and parylene C cracking scores. In Monkey U, the Mann-Whitney U test resulted in significant p-values for all scores, while the Levene test resulted in an insignificant p-value for abnormal debris, tip breakage, and coating cracks scores. Finally, in Monkey C, the Mann-Whitney U test resulted in an insignificant p-value for parylene C delamination and parylene C cracking scores, while the Levene test resulted in an insignificant p-value for abnormal debris, parylene C delamination, and parylene C cracking scores.’

To point out another example, this chunk of text is highly repetitive and is unnecessary, as the reader can simply refer to Supplementary Table 4. It should be completely rephrased and summarized, to deliver the key message, i.e. briefly describe what kinds of damage occurred for which arrays. Also, what is the point of the two statistical tests? What are the authors trying to conclude?

Thank you for this recommendation. We have rephrased and pared down the text to reflect this note.

(13) Discussion: ‘Similarly, other work did not show significant differences in SEM-visible degradation between both platinum and iridium oxide coated electrodes used for stimulation [24, 25].’ What differences are being referred to here? Differences in degradation between stimulated Pt versus stimulated IrOx electrodes? Or between stimulated Pt and unstimulated PT electrodes? Stimulated IrOx and unstimulated IrOx? Or something else?

Thank you for your questions. We are comparing platinum against iridium oxide in this sentence. The wording of our original text has been updated to clarify our intention.

(14) Supplementary Tables: P-values lower than .05, .01, and .001 should simply be replaced with ¡.05, ¡.01, and ¡.001. The alpha value after a Bonferroni correction should be stated somewhere in each table or table caption.

Thank you for this recommendation. We have edited the tables to reflect this note.

(15) Title: ‘Material Damage to Multielectrode Arrays after Electrolytic Lesioning is in the Noise’ I don’t understand what the title means. What is in the noise? And what is ‘the noise’?

“In the noise” is a colloquialism referring to how background information (“noise”) may obscure or distract from other features. This title conveys how material damage to multielectrode arrays due to electrolytic lesioning is largely obscured by the general damage observed on multielectrode arrays after implant and explant.

(16) This reference has been left out altogether: Chen et al., 2014. The effect of chronic intracortical microstimulation on the electrode-tissue interface.

Thank you, this reference is now included.

**Reviewer #2 (Recommendations for the authors):**

(1) The number of lesion electrodes is low, especially since there are only 2-10 lesion electrodes on three of the four arrays, yielding limited statistical power.

We agree that the low number of lesioned electrodes limits statistical power. However, due to ethical considerations, it is unlikely for arrays to contain much more than this number of lesion electrodes.

(2) The dataset includes both platinum and iridium oxide-coated electrodes. A direct comparison of their damage profiles would be informative.

Thank you for this recommendation. We have included this additional analysis in Supplementary Materials.

(3) It is unclear what “is in the Noise” in the title means without reading the manuscript. It is helpful to improve the clarity of the title.

Thank you for this recommendation.

(4) Please spell out “PMd” and “M1” at first mention to facilitate reading.

Thank you for this note. The text has been updated to reflect this recommendation.
